# Integrating AI into undergraduate medical education: an exploration of learner-centered approaches through AI

**DOI:** 10.3389/fmed.2026.1824069

**Published:** 2026-05-22

**Authors:** Blaine A. Jacobs, Leslie Cano, Jessica M. Bradley

**Affiliations:** 1 Department of Applied Biomedical Sciences, University of the Incarnate Word School of Osteopathic Medicine, San Antonio, TX, United States; 2 Office of Faculty Development, University of the Incarnate Word School of Osteopathic Medicine, San Antonio, TX, United States

**Keywords:** artificial intelligence, integrative curriculum, learner-centered, patient-based simulation, undergraduate medical education

## Abstract

**Introduction:**

Advances in artificial intelligence (AI) offer promising opportunities to enrich medical education by supporting self-directed learning, providing personalized feedback, and simulating clinical and scientific reasoning. Although AI use has expanded in graduate medical education for clinical decision training and administrative support, its effectiveness in undergraduate medical education remains insufficiently established. Empirical data examining how AI instruction influences academic performance, knowledge consolidation, engagement, and critical thinking are limited. This study evaluated whether incorporating AI-patient-based simulations into classroom activities enhances learning outcomes and student experience within an integrated osteopathic medical school curriculum.

**Methods:**

Using a mixed-methods survey design, quantitative and qualitative data were collected and analyzed to examine students’ perceptions of faculty-developed AI-patient-based simulations integrated into classroom instruction.

**Results:**

Students reported that AI-patient-based simulations supported engagement, reinforced learning, and facilitated application of clinical reasoning concepts. Qualitative findings suggested that the simulations promoted active participation and encouraged self-directed learning while highlighting the importance of faculty guidance in interpreting AI-generated content.

**Discussion:**

Overall, findings suggest that AI-patient-based simulations can provide meaningful support to medical student learning when thoughtfully integrated into the curriculum and accompanied by explicit instruction regarding limitations, ethical considerations, and responsible AI use. Further research is needed to determine the long-term effects of AI-assisted instruction on academic performance and critical thinking development.

## 1 Introduction

Recent advances in artificial intelligence (AI), particularly in natural language processing and adaptive learning platforms, have opened new opportunities for enhancing medical education. Studies have shown that AI can support self-directed learning and promote engagement through interactive learning environments (1, 2). AI tools can provide personalized feedback, simulate clinical reasoning, and adapt to individual student performance in real time.

Despite these promising developments, insight into the integration of AI into undergraduate medical education remains limited, with most studies focusing on clinical decision support or administrative applications rather than foundational classroom learning. Current evidence suggests that students are receptive to AI-assisted education but lack sufficient exposure or training in using such tools effectively (3, 4). Furthermore, little empirical research has directly compared AI-integrated instructional methods with traditional didactic teaching in regard to their impact on knowledge consolidation, engagement, and academic outcomes in medical students. This evidence gap limits our understanding of how AI may influence essential competencies, such as critical thinking, self-regulated learning, and clinical reasoning.

The purpose of this study was to develop AI-patient-based simulations that present students with clinical scenarios to assess physiology and pharmacology concepts in the classroom. We also aimed to provide students with exposure to AI while fostering critical evaluation of AI-generated responses. We explored whether the use of AI patient simulations enhanced students’ understanding of class material, ability to apply knowledge, and classroom engagement. Additionally, we examined students’ perceptions of AI in the classroom and its perceived reliability.

## 2 Materials and methods

Using a mixed-methods survey design, we collected and analyzed quantitative and qualitative data to examine students’ perceptions of integrating AI tools in a classroom setting. This allowed for an assessment of students’ overall impressions, as well as perceived benefits and challenges related to AI-patient-based simulations in class.

### 2.1 Description of AI simulations for classroom use

The AI simulations were created by Bradley and Jacobs using Microsoft Co-Pilot (GPT-5) web experience^1^ through the University of the Incarnate Word (UIW) access. During class, students were asked to complete informed consent forms prior to being provided with a link for the simulation.

The students were asked to activate one of the following agents or simulations:

- AI Simulation: Heart Failure and Chronic Kidney Disease
- AI Simulation: Differential Diagnosis and Treatment of Syndrome of Inappropriate Anti-Diuretic Hormone (SIADH) vs. Diabetes Insipidus
- AI Simulation: Gout Management

In the simulations, the AI software generated randomized simulated patients that varied with each student, and each time the simulation was repeated. All simulations were built using specific prompts and based on the learning objectives assigned for the session. Agents were instructed not to give students answers or hints until they attempted to answer the prompt.

One of the design limitations was the inability to share the simulations we created with students due to copyright restrictions. We realized that only publicly available websites could serve as knowledge sources for Microsoft Co-Pilot Agents at UIW, and that UIW textbook resources were not an option. Therefore, to ensure content accuracy, we utilized the following publicly available websites:

- Tulane University School of Medicine’s Pharmwiki.^2^
- NIH’s National Library of Medicine: StatPearls Physiology, Endocrine Hormones.^3^

#### 2.1.1 AI simulation: heart failure and chronic kidney disease

The heart failure and chronic kidney disease simulation was developed for second-year osteopathic medical students (OMS) II as part of their combined cardiovascular and renal unit. It was presented as a workshop after class to show students how to create and use their own simulators using Microsoft Co-Pilot for self-directed learning. Students were instructed to create a simulator with the same patient profile, core functionality, and simulation rules described below.

The instruction prompts:

- Simulate a 65-year-old male patient with long-standing hypertension, type 2 diabetes, ischemic cardiomyopathy (EF 35%), mild chronic kidney disease (eGFR ~50 mL/min), and no known drug allergies.
- Present the patient with progressive shortness of breath, orthopnea, 2 + bilateral lower extremity edema, and mild weight gain over the past week.
- Provide vital signs: BP 158/92, HR 92, RR 20, O₂ sat 93% on room air.
- On exam, include crackles in lung bases, S4 gallop, and pitting edema at ankles.
- Labs: Na^+^ 138, K^+^ 3.4, Cl^−^ 101, HCO₃^−^ 28, BUN 30, Cr 1.6 (baseline 1.2), Glucose 150 mg/dL.
- Respond to student clinical decisions by updating signs, symptoms, and labs, simulating improvement or deterioration based on choices.
- Focus the simulation on diuretic management and related clinical reasoning.
- Prompt students to explain basic science knowledge related to the case before proceeding.
- Never provide direct answers, even if asked; always wait for the student to attempt an answer first.
- Remain in character as the AI patient simulator at all times.
- Begin the simulation only when prompted with “Start the patient simulation.”
- Challenge poor decisions by simulating clinical deterioration and provide realistic feedback.
- Do not break character or provide meta-commentary about the simulation process.

#### 2.1.2 AI simulation: differential diagnosis and treatment of syndrome of inappropriate anti-diuretic hormone vs. diabetes insipidus

The SIADH and Diabetes Insipidus simulation was utilized in our Endocrine Unit for OMS-IIs. The goal for this simulation was to produce an interactive simulator for medical students to consolidate endocrine physiology and pharmacology by simulating patient cases involving SIADH, nephrogenic diabetes insipidus, or central diabetes insipidus. Students were to interpret lab findings, make diagnoses, and determine pharmacologic management through multiple-choice questions.

The instruction prompts:

- Simulate a patient with SIADH, nephrogenic diabetes insipidus, or central diabetes insipidus, randomly selecting one for each session.
- Present laboratory findings and diagnostic tests for interpretation.
- Prompt students with multiple-choice questions based on session objectives:
  - Explaining pathophysiology, presenting features, diagnosis, and treatment of hyper- and hyposecretory states of antidiuretic hormone.
  - Distinguishing between central and nephrogenic diabetes insipidus based on plasma ADH levels and response to ADH injection.
- Always stay in character as the AI patient simulator.
- Do not give answers or hints to questions, even when asked.
- Do not show answers in tabs at the bottom
- Wait for the student to attempt an answer before responding.
- Only provide feedback at the end of the simulation, not after each answer
- Begin the simulation only when prompted with “Start the patient simulation.”

#### 2.1.3 AI simulation: gout management

The gout management simulation was part of the Musculoskeletal Unit for our OMS-Is. The goal of this simulation was for students to relate the patient’s clinical presentation and the stages of gout to the appropriate pharmacological treatment.

The instruction prompts:

- Simulate patients with various stages of gout, presenting them as different individuals.
- Relate the physiology and pathology of gout to pharmacology in each case.
- Present patient cases with lab results and histories included when pertinent.
- Generate NBOME-style multiple choice quizzes with 5 answer choices and one correct answer, focused on basic science.
- Ask 10 questions per session before grading and providing rationales for each answer.
- Do not prompt, hint, or suggest correct answers at any point during quizzes.

### 2.2 Participation and data collection

For each simulation, students were invited to complete an anonymous, voluntary survey following participation in an AI-integrated learning activity. The survey did not collect names, student identification numbers, or any other personally identifiable information.

The survey included:

- Likert-scale items assessing perceived usefulness, engagement, learning support, confidence, and overall experience with AI tools
- Open-ended questions inviting students to describe their experiences, perceived benefits, and challenges related to AI use

### 2.3 Quantitative analysis

Responses to Likert-scale items were analyzed using descriptive statistics. Means, standard deviations, and medians were calculated to summarize student perceptions across survey items. Quantitative analyses were performed using Qualtrics.

### 2.4 Qualitative analysis

Open-ended survey responses were analyzed using inductive thematic analysis. All responses were reviewed to identify recurring ideas and patterns. Initial codes were generated directly from students’ language rather than predefined categories. Human reviewers conducted all coding and interpretation. Codes were grouped into broader themes related to engagement, perceived usefulness of AI tools, and barriers to adoption. Differences in interpretation were resolved through discussion until consensus was reached.

### 2.5 Ethical considerations

The University of the Incarnate Word Institutional Review Board reviewed and approved the study protocol (no. 2025-1803-NRR-v3.7283). It was determined that the study did not meet the federal regulatory requirements for human subjects research under 45 CFR 46.102(d) and was categorized as not human subjects research. The confidentiality of the study participants’ identities was maintained throughout the study by keeping their information confidential. This was a voluntary and non-compensated survey, and all participants were asked to provide their consent via the Qualtrics link before completing the remaining survey responses.

### 2.6 Sample size

With a sample size of 39 students and an observed standard deviation of approximately 1.0, the mean Likert-scale responses can be estimated with a 95% confidence interval half-width of approximately ±0.31. The sample size of 39 students is sufficient for the descriptive aims of the study, providing reasonable precision for estimation of Likert-scale responses and supporting inductive thematic analysis of open-ended responses.

### 2.7 Inclusion and exclusion

Inclusion criteria include current enrollment in the selected medical course(s), consent to participate, and access to the technology required to interact with AI tools.

Students will be excluded if they do not provide informed consent and/or fail to complete key components of the course.

### 2.8 Analysis

Student perceptions of AI integration collected via surveys were analyzed using:

1. Descriptive statistics of Likert scale item frequencies (Table 1).
2. Thematic analysis for open-ended responses to identify common attitudes, concerns, and perceived benefits or barriers (Table 2).

**Table 1:** Descriptive statistics for Likert-scale survey items.

Item	Survey construct	Mean	SD	Median
Q1_1	AI tools were useful for my learning	4.41	0.63	4
Q1_2	AI tools increased my engagement	3.92	0.97	4
Q1_3	AI tools supported understanding of course material	4.33	0.65	4
Q1_4	AI tools helped apply concepts	3.95	0.90	4
Q1_5	AI tools enhanced overall learning experience	4.13	0.76	4
Q1_6	I trust the accuracy of AI-generated responses	3.72	1.36	4
Q1_7	I would recommend AI tools for learning	4.28	0.90	5

**Table 2:** Codebook for thematic analysis of open-ended responses.

Theme	Description	Excerpt or codes
Tool for active learning, practice and feedback	AI facilitated engagement with class content through practice questions and formative feedback.	“*It gives feedback and allows for application of material*.” “*I think it helped to make it interactive*.”
Organizing and synthesizing course content	AI allowed for organizing information, notes, outlining key concepts, consolidating class readings, and identifying relevant research topics.	“*AI allows it to organize the information in a table or in a neat summary that makes the information easier to understand*.”
Interactive questioning and clarification	AI functioned as a low stakes responsive tool for asking questions and clarifying understanding.	“*AI allowed for asking questions and getting quick answers*.”
Trust, reliability, and ethical concerns	Students’ use of AI was shaped by concerns about response accuracy, reliability, and verification.	“*Sometimes it gives incorrect information*,” “*I sometimes get worried that I will not be able to verify if the information is correct*,”
Quality of user input and interpretation	Successful use of the AI tool depends on the user’s ability to engineer prompts and agent instructions	“*You have to know how to ask the question correctly*.” “*Setting up the program and making it understandable was difficult*.”
AI as a supplemental resource	AI served as a supplemental resource alongside class content and readings provided additional practice and clarification.	“*It helped but I still relied on class materials*.” “*I used it alongside my notes*”

Qualitative data analysis (e.g., student reflections and open-ended survey responses) was analyzed using thematic analysis. Responses were coded inductively to identify common themes related to engagement, perceived usefulness of AI tools, and barriers to adoption. Coding was conducted by two independent reviewers to enhance reliability, and discrepancies were resolved through discussion.

## 3 Results

### 3.1 Descriptive statistics

A total of 39 student responses were included in the quantitative analysis. Descriptive statistics were calculated for seven Likert-scale items assessing perceptions of AI-integrated learning, including usefulness, engagement, learning support, and confidence.

Overall, students reported favorable perceptions of AI integration. Mean scores across items ranged from 3.72 to 4.41 on a five-point Likert scale, with median responses of 4 (“Agree”) or 5 (“Strongly Agree”) for all items. The highest mean score was observed for perceived usefulness of AI tools (M = 4.41, SD = 0.63), while items related to confidence and trust demonstrated greater variability (SD up to 1.36).

These findings indicate that students generally perceived AI tools as beneficial to their learning experience, with some heterogeneity in confidence regarding accuracy and reliability.

### 3.2 Thematic analysis

Open-ended survey responses were analyzed using thematic analysis. Thematic analysis of student responses revealed six interrelated themes describing how students experienced the use of AI tools in the classroom.

#### 3.2.1 Theme 1: Tool for active learning, practice, and feedback

Students described the AI tool as enhancing active learning with course content by providing opportunities for self-directed practice and immediate feedback. As one student reflected, “*it gives feedback and allows for application of material*,” while another observed, that “it helped to make it interactive”, demonstrating how AI supported both comprehension and participatory learning.

#### 3.2.2 Theme 2: Organizing and synthesizing course content

Students described AI tools as supporting their understanding of course materials by organizing and synthesizing information, making complex content more accessible. The structured formats provided by AI help to clarify key concepts and eased the cognitive load of processing large volumes of information. As one student explained, “*AI allows it to organize the information in a table or in a neat summary that makes the information easier to understand*,” highlighting how AI facilitated comprehension and streamlined course content.

#### 3.2.3 Theme 3: Interactive questioning and clarification

Students described AI as a low-stakes, responsive tool that supported their learning by allowing them to ask questions and clarify their understanding in real time. The technology provided immediate feedback, making it easier for students to explore ideas without fear of judgement. One student noted, “*AI allowed for asking questions and getting quick answers*,” illustrating the value of rapid, accessible guidance. Overall, this theme captures AI’s potential to foster an engaging, supportive environment for self-directed learning.

#### 3.2.4 Theme 4: Trust, reliability, and verification

While perceptions of AI use were mostly positive, students expressed concerns about the accuracy and reliability of its responses. Several students emphasized the need to verify outputs, noting that “*sometimes it gives incorrect information*,” while another shared, “*I sometimes get worried that I won’t be able to verify if the information is correct*,” highlighting anxiety around trustworthiness. Students also cited hallucinations, defined as instances where AI generates false or misleading information, as a challenge to adoption. This theme emphasizes the importance of cultivating both evaluation skills and ethical awareness when using AI for learning.

#### 3.2.5 Theme 5: Quality of user input and interpretation

Students emphasized that successful AI use depended on the clarity of their prompts and the accuracy of the input provided. Many reported challenges in formulating questions, interpreting outputs, or assessing the trustworthiness of AI-generated information. As one student explained, “*You have to know how to ask the question correctly*,” and another student shared, “*Setting up the program and making it understandable was difficult*.” These challenges highlight the effort required to navigate AI effectively. These responses suggest that effective AI use requires both technical skill and conceptual understanding of the content.

#### 3.2.6 Theme 6: AI as a supplement rather than a replacement

Students described AI as a supportive, supplementary learning resource that enhanced their engagement with class materials without replacing traditional instruction, faculty guidance, or primary resources. One student noted, “*it helped but I still relied on class materials*,” while another explained, “*I used it alongside my notes and other resources*.” This theme demonstrates AI’s role as an adjunct rather than a replacement for traditional instruction. Students value AI for providing additional practice, clarification, and accessible feedback, reinforcing key concepts from class sessions and readings.

## 4 Discussion

Recent studies have demonstrated growing interest in integrating AI into medical education, with reported positive learner perceptions and potential benefits for critical thinking and self-directed learning (5). Prior work has described the use of AI-generated treatment plans, problem-based learning activities, and elective coursework, suggesting that AI can function as a valuable educational adjunct when used appropriately (6–8). These findings support the premise that AI should be intentionally integrated into medical curricula with clear andragogical goals rather than employed as an unguided information source. Accordingly, faculty engagement is essential to ensure that learners understand both the capabilities and limitations of AI and are taught to use these tools responsibly and critically.

The development of AI-patient-based simulations at our institution was driven by the integrated structure of our pre-clinical curriculum, in which sessions are co-facilitated across disciplines, such as physiology and pharmacology (9). In considering ways to use AI in the classroom, we found that externally developed or generic AI tools were insufficient to meet session specific learning objectives. Building simulations internally allowed us to align AI activities directly with established curricular goals while preserving the coherence and integrative nature of existing sessions. This approach ensured that AI was embedded as a complementary instructional strategy rather than an isolated technological addition.

Agent-based AI simulations were selected over simple chat interfaces since clinical reasoning is fundamentally a performance-based skill. Unlike chat-based systems that may encourage passive consumption of information, agent-based simulations require students to actively engage in clinical reasoning by asking questions, selecting diagnostic tests, committing to management decisions, and experiencing the consequences of those decisions. This structure mirrors authentic clinical encounters and allows students to learn through safe failure, immediate feedback, and iterative correction. Additionally, the use of curated, faculty-provided knowledge sources within the agents helped mitigate concerns related to AI hallucinations and inaccuracies. Leveraging Microsoft Copilot further allowed simulations to remain securely accessible within the institutional network while maintaining curricular alignment.

Despite careful planning, the implementation of AI-patient-based simulations presented several logistical challenges. Initial efforts using ChatGPT were abandoned given that custom simulations could not be shared due to restrictions that prohibit giving medical advice. Although transitioning to Microsoft Copilot improved institutional compatibility, additional barriers emerged, including organizational permission settings and access limitations when agents relied on faculty-only or licensed resources. These challenges necessitated adaptive strategies to maintain educational objectives while navigating platform constraints. As an interim solution, a faculty-led workshop was developed to teach students how to build a choose-your-own adventure AI-patient simulator within Microsoft Copilot that allowed them to explore multiple clinical pathways and observe variable outcomes based on their decisions.

Once sharing barriers were resolved, AI-patient-based simulations were successfully implemented as in-class activities. At this stage, the instructional design shifted from a choose-your-own-adventure format to a fixed-path structure. Fixed-path simulations were used for topics such as SIADH, diabetes insipidus, and gout management, presenting standardized cases with laboratory data and multiple-choice questions targeting physiology and pharmacology concepts. Although this format limited student agency, it reduced variability, increased reproducibility, and better accommodated time constraints inherent to classroom instruction. The selection of simulation format was therefore guided by instructional context rather than technological preference.

Consistent with other reports, our student feedback indicated generally positive perceptions of AI-integrated learning experiences (10, 11). Quantitative survey results demonstrated a high level of perceived usefulness and engagement, while qualitative responses highlighted AI’s role in clarifying and reinforcing complex concepts. At the same time, students expressed concerns regarding the accuracy and reliability of AI-generated information, including awareness of hallucinations and incorrect outputs. Importantly, this cautious stance suggests that students were not engaging with AI uncritically, which is an advantageous perspective for developing medical professionals. These findings underscore the importance of explicit instruction in AI literacy, including effective prompting strategies and critical evaluation of AI responses.

## 5 Limitations

To our best knowledge, this is one of the first papers that discusses the development and utilization of custom faculty-built, AI-patient-based simulation in undergraduate medical education. Utilizing a mixed-method study design, we gathered valuable insights from student perspectives that reveal the use of these simulations have meaningful impact on learning. However, we also acknowledge certain limitations within our study. First, the research was conducted exclusively in one university. Our goal was to build these simulations to fit into our unique, integrated curriculum although this may have impacted the generalization of our findings. Additionally, the small sample size may have introduced selection bias as those students that opted in to the study have more experience, motivation, or interested in learning AI, skewing the self-reported perceptions in a positive manner.

Secondly, while the qualitative data provided insight into student experiences, the use of survey-based responses limited opportunities for deeper probing and clarification that are characteristic of qualitative inquiry. Further research could build on these findings through more in-depth qualitative approaches, such as interviews or focus groups, to further explore the nuances of student experiences and perspectives with AI.

## 6 Conclusion

Collectively, these findings suggest that AI-patient-based simulations can meaningfully support medical student learning when thoughtfully integrated into the curriculum and accompanied by explicit guidance on limitations and responsible use. Faculty-designed simulations that align with curricular objectives and emphasize critical engagement may help maximize educational value while minimizing risks associated with AI misuse. However, the major limiting factor that is noted across other AI studies is the ever growing and evolving nature of this technology and the lack of knowledge and experience we have to stay current. With this, it is important for faculty to have at least a basic knowledge of its use, to support students as they turn to these platforms to aid their learning. Future work will focus on evaluating the impact of AI simulations on academic performance and developing additional instructional activities that further strengthen students’ ability to critically appraise AI-generated information.

## Data Availability

The raw data supporting the conclusions of this article will be made available by the authors, without undue reservation.

## Footnotes

1
https://copilot.microsoft.com

2
https://tmedweb.tulane.edu/pharmwiki/doku.php/start

3
https://www.ncbi.nlm.nih.gov/books/NBK538498/
